# A general metal-free approach for the stereoselective synthesis of *C*-glycals from unactivated alkynes

**DOI:** 10.3762/bjoc.10.277

**Published:** 2014-11-12

**Authors:** Shekaraiah Devari, Manjeet Kumar, Ramesh Deshidi, Masood Rizvi, Bhahwal Ali Shah

**Affiliations:** 1 Academy of Scientific and Innovative Research (AcSIR); Natural Product Microbes, CSIR-Indian Institute of Integrative Medicine, Canal Road, Jammu -Tawi, 180001, India; 2 Department of Chemistry, University of Kashmir, 190006, India

**Keywords:** α-selective, *C*-alkynylation, glycal, metal free, TMSOTf

## Abstract

A novel metal-free strategy for a rapid and α-selctive *C*-alkynylation of glycals was developed. The reaction utilizes TMSOTf as a promoter to generate in situ trimethylsilylacetylene for *C*-alkynylation. Thanks to this methodology, we can access *C*-glycosides in a single step from a variety of acetylenes , i.e., arylacetylenes and most importantly aliphatic alkynes.

## Introduction

**Scheme 1:**
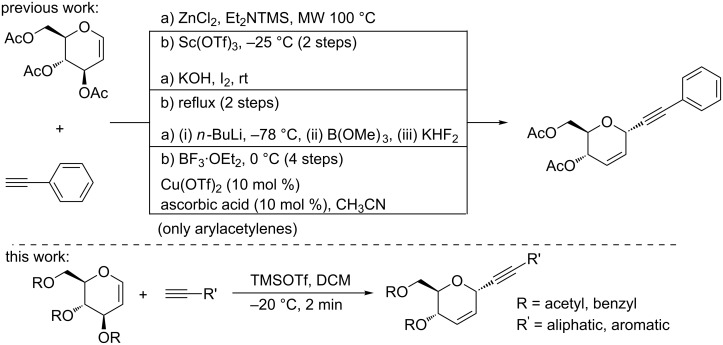
*C*-alkynylation of glycals.

*C*-Glycosides represent an important class of carbohydrate mimics, owing to their presence in a large number of biologically active natural products, such as palytoxin, spongistatin, vitexin, orientin, bergenin and halichondrin [1–5]. Over the years myriad methods have appeared for their efficient and stereoselective synthesis [6–12]. Among these methods, *C*-alkynylation [13–16] is of particular interest as it is amenable to further modifications into chiral molecules, carbohydrate analogues, and natural products, such as tautomycin [17–18] and ciguatoxin [19–21]. In recent past, significant efforts have been directed toward the *C*-alkynylation of glycals [9,22–23]. However, all these methods use priorly activated terminal alkynes, e.g., silylacetylene, activated with various Lewis acids such as SnCl_4_, BF_3_·OEt_2_, TiCl_4_, I_2_, InBr_3_, and ZrCl_4_ [24–30], followed by a Ferrier type rearrangement [31–34] (Scheme 1). A consequence of the prior activation of alkynes is the involvement of multiple steps and thus loss of yields. Recently, Mukherjee and co-workers [35] have reported a one-step *C*-alkynylation of glycals by using metal in combination with a co-oxidant, i.e., Cu(OTf)_2_ with ascorbic acid. However, a drawback of the method is its limited applicability to arylacetylenes only and its restricted selectivity to reactions performed in the solvent acetonitrile (Scheme 1).

It is obvious from the preceding discussion that the stereoselective addition of alkynes to the anomeric carbon of sugar nuclei in a single step still represents an interesting challenge. We reasoned that the development of a strategy which in situ activates the terminal alkyne and further catalyzes the reaction without the aid of other Lewis acids might be a solution to this problem. Thus, in continuation of our efforts [36–38], we describe a highly stereoselective TMSOTf catalyzed rapid *C*-alkynylation of glycals with a wide variety of unactivated alkynes, i.e., arylacetylenes and aliphatic alkynes. The method circumvents the use of metal catalysts/co-oxidants and exhibits short reaction times, i.e., 2 min. This method may find use in a large number of reactions, which are characterized by a requirement of pre-formed trimethylsilylacetylene.

## Results and Discussion

Initial investigations involved the use of 3,4,6-tri-*O*-acetyl**-**D**-**glucal (**1**) and phenylacetylene (**2**) as model substrates with TMSOTf as a promoter within DCM at −20 °C. To our delight TLC showed full consumption of the starting materials in 2 min and yielded the desired product **3a** in 80% yield (Table 1, entry 1). The structure and stereochemistry were elucidated by a comparison of the chemical shifts to that of reported values [35]. Also, the stereochemistry of the resulting product **3a** was unambiguously established as α by NOESY spectra, indicating cross peaks between H-1, H-6 and H-4. To further establish the role of the catalyst we repeated the reaction with other Lewis acid catalysts, such as In(OTf)_3_, Cu(OTf)_2_, Sc(OTf)_3_ and BF_3_·OEt_2_, but no product could be obtained (Table 1, entries 2–5). Next, we focused our attention on optimizing the suitable amount of the catalyst loading. We observed that a decrease of the catalyst loading below 30 mol % led to no product formation even after 1 h (Table 1, entries 6 and 7). However, an increase to 40 mol % with longer reaction time resulted in a loss of yield due to the degradation of the product (Table 1, entry 8). A further increase in catalyst loading had no significant impact on the overall reaction yield and time (Table 1, entry 9). We also examined the effect of temperature on the reaction, and observed that both an increase (up to rt) and a decrease (to −40 °C) resulted in the loss of yield (Table 1, entries 10–13).

**Table 1:** Alkynylation reaction of 3,4,6-tri-*O*-acetyl-D-glucal with phenylacetylene^a^.

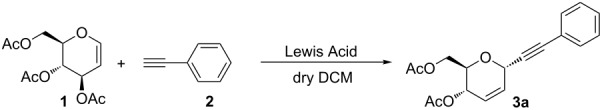						

entry	Lewis acid	equiv	*T* (°C)	time	yield^b^	(α/β)^c^

1	TMSOTf	0.5	−20	2 min	80	99:1
2	In(OTf)_3_	0.5	−20	5 h	–	–
3	Cu(OTf)_2_	0.5	−20	5 h	–	–
4	Sc(OTf)_3_	0.5	−20	5 h	–	–
5	BF_3_·OEt_2_	0.5	−20	5 h	–	–
6	TMSOTf	0.2	−20	1 h	–	–
7	TMSOTf	0.3	−20	1 h	<10	ND
8	TMSOTf	0.4	−20	15 min	57	ND
9	TMSOTf	0.8	−20	2 min	82	ND
10	TMSOTf	0.5	−40	5 min	63	ND
11	TMSOTf	0.5	−10	2 min	77	ND
12	TMSOTf	0.5	0	2 min	59	ND
13	TMSOTf	0.5	rt	2 min	37	ND

^a^In all cases 1 equiv of **1** and 1.2 equiv of **2** were used; ^b^isolated yields; ^c^determined by ^1^H, ^13^C NMR and NOESY spectra; ND = not determined.

**Scheme 2:**
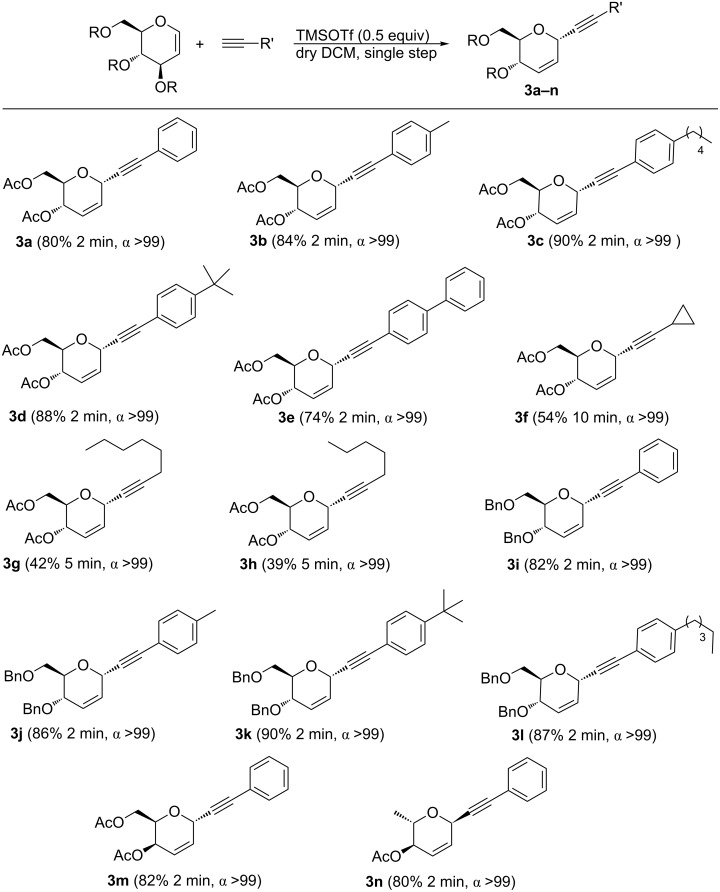
Substrate scope of the reaction process under optimized reaction conditions.

The scope of the present method was further expanded to a variety of alkynes and glycals (Scheme 2). It was established that the system was tolerant to a wide variety of electron-donating as well as electron-withdrawing terminal alkynes to give the corresponding products **3a–e** in excellent yields and selectivity. It is noteworthy that the earlier reported [35] single-step strategy failed to yield a product with aliphatic alkynes, so that we applied the method to aliphatic alkynes. The reaction with cyclopropylacetylene, hept-1-yne and oct-1-yne maintained a high selectivity and gave the corresponding products **3f–h** in 54, 42 and 39% yield, respectively. To further broaden the scope of the reaction, tri-*O*-benzyl-D-glucal was subjected to the reaction with phenylacetylene, *p*-methylphenylacetylene, *p*-(*tert*-butyl)phenylacetylene and *p*-pentylphenylacetylene giving the corresponding products **3i–l** in 82, 86, 90, and 87% yield, respectively, with >99% selectivity. Also, the reaction with other glycals, i.e., 3,4,6-tri-*O*-acetyl**-**D**-**galactal and 2,4-di-*O*-acetyl**-**L**-**rhamnal with phenylacetylene gave the corresponding products **3m** and **3n** in 82 and 80% yield, respectively, and with a high selectivity.

**Scheme 3:**
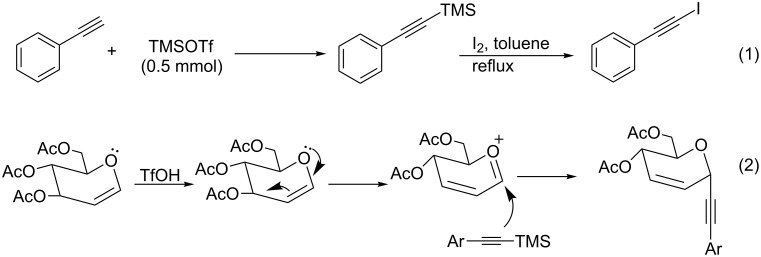
Plausible mechanism of the Ferrier rearrangement.

The present results indicate the activation of terminal alkynes by TMSOTf forming trimethylsilylacetylenes [39]. In order to confirm the formation of trimethylsilylacetylenes, we attempted a control experiment involving the addition of molecular iodine instead of glucal. As expected [40–41], the reaction on heating at 70 °C for 3 h gave the iodinated phenylacetylene (Scheme 3, reaction 1 & Figure S1, Supporting Information File 1). Thus, the triflic acid generated in situ consequent to the formation of trimethylsilylacetylene activates the tri-*O*-acetyl**-**D**-**glucal forming an oxonium ion intermediate, which is attacked by trimethylsilylacetylene to give the corresponding product (Scheme 3, reaction 2). The stereochemistry of the reaction products is possibly determined by the coordination between two π-electron orbitals of the oxocarbonium ion and the acetylene groups, while the stereoelectronic control allows the α-pseudo-axial orbital to form the bond [35].

## Conclusion

In conclusion, we developed a highly efficient and α-selective method for the synthesis of alkynyl glycosides from virtually any alkyne, that is, aliphatic and aromatic. To the best of our knowledge, this is the first report which descibres the in situ generation of trimethylsilylacetylene and its subsequent usage for *C*-alkynylation without the co-addition of a Lewis acid. The protocol may find application in a large number of reactions catalyzed by Lewis acid wherein pre-formed silylated terminal alkynes are required. Further studies are underway to broaden the scope of the present reaction.

## Supporting Information

File 1 Experimental procedures, characterization data, and ^1^H and ^13^C NMR spectra of relevant compounds.
