# Supplementary material for: A general metal-free approach for the stereoselective synthesis of C-glycals from unactivated alkynes
Source: Beilstein J Org Chem. 2014 Nov 12;10:2649–53. doi: 10.3762/bjoc.10.277 (PMC4273296; doi:10.3762/bjoc.10.277)
Supplement: File 1 — Experimental procedures, characterization data, and 1H and 13C NMR spectra of relevant compounds. [file Beilstein_J_Org_Chem-10-2649-s001.pdf]

Supporting Information

for

A general metal-free approach for the stereoselective synthesis of *C*-glycals from unactivated alkynes

Shekaraiah Devari[1], Manjeet Kumar[1], Ramesh Deshidi[1], Masood Rizvi[*,2] and Bhahwal Ali Shah[*,1]

Address: [1]Academy of Scientific and Innovative Research (AcSIR); Natural Product Microbes, CSIR-Indian Institute of Integrative Medicine, Canal Road, Jammu -Tawi, 180001, India and [2]Department of Chemistry, University of Kashmir, 190006, India

Email: Masood Rizvi - masoodku2@gmail.com; Bhahwal Ali Shah - bashah@iiim.ac.in

*Corresponding author

Experimental procedures, characterization data, ^{1}H and ^{13}C NMR spectra of relevant compounds

General Methods

^{1}H and ^{13}C NMR spectra were recorded on 400 and 500 MHz spectrometer with TMS as an internal standard. Chemical shifts are expressed in parts per million (δ ppm), *J* values are given in hertz. Mass spectra were recorded on 4800 MALDI-TOF-TOF Analyzer. Infrared spectra were recorded on a Perkin Elmer FT-IR spectrophotometer. Reagents and solvents used were mostly AR grade. Silica gel coated aluminum plates from M/s Merck were used for TLC.

Typical Procedure C-alkynylation of Glycals

To a mixture of glucal (1 equiv) and alkyne (1.2 equiv) in DCM at −20 °C, was added TMSOTf (50 mol %) and kept on stirring till completion of reaction as monitored on TLC. The reaction was quenched with sat. $NaHCO_3$ solution and extracted with DCM (twice), washed with sat. brine and purified over silica gel (100–200 mesh) using hexane:EtOAc as eluent to afford pure products**.**

List of known compounds and their references:

Entry	Compounds	Reference
1	**3a, 3b, 3d, 3f, 3g**	*Org. Lett.* **2008,** *10*, 5215-5218.
2	**3c, 3e, 3i, 3m, 3n**	*Chem. Commun***. 2013**,*49***,** 10154-10156.
3	**3h**	*Tetrahedron Lett.* **2006,** *47*, 5269-5272.

Spectral Data:

Compound 3a

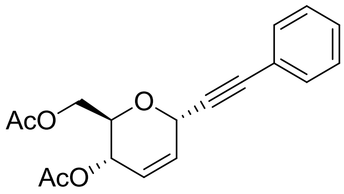

^{1}H NMR (500 MHz, $CDCl_3$) δ 7.48 – 7.42 (m, 2H), 7.37 – 7.29 (m, 3H), 5.99 (ddd, *J* = 10.1, 3.3, 1.7 Hz, 1H), 5.84-5.82 (t, *J* = 13.4 Hz, 1H), 5.35 (dd, *J* = 8.9, 1.8 Hz, 1H), 5.21 (d, *J* = 1.6 Hz, 1H), 4.26 (t, *J* = 8.1 Hz, 2H), 4.23 – 4.18 (m, 1H), 2.10 (t, *J* = 6.7 Hz, 6H, Ac); ^{13}C NMR (125 MHz, $CDCl_3$) δ 170.9, 170.3, 131.8, 129.2, 128.8, 128.3, 125.5, 122.2, 86.7, 84.7, 70.0, 64.8, 64.5, 63.1, 21.0, 20.8; HRMS: calcd for $C_{18}H_{18}O_5$ $[M+Na]^+$ 337.1047, found 337.1057. IR ($CHCl_3$): 3054, 2955, 2358, 1745, 1236 cm^{-1}. $[\alpha]_D^{23}$= -58.2^o (c = 0.5 $CHCl_3$).

Compound 3b

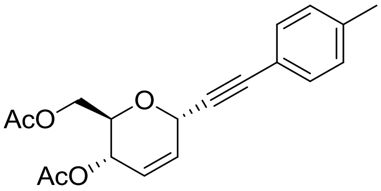

^{1}H NMR (400 MHz, $CDCl_3$) δ 7.34 (d, *J* = 8.1 Hz, 2H), 7.13 (d, *J* = 7.9 Hz, 2H), 5.98 (ddd, *J* = 10.2, 3.5, 1.9 Hz, 1H), 5.86 – 5.78 (m, 1H), 5.34 (dd, *J* = 8.9, 1.9 Hz, 1H), 5.22 – 5.18 (m, 1H), 4.26 (t, *J* = 5.2 Hz, 2H), 4.23 – 4.17 (m, 1H), 2.35 (s, 2H), 2.12 – 2.09 (s, 6H); ^{13}C NMR (100 MHz, $CDCl_3$) δ 170.9, 170.3, 139.0, 131.7, 129.4, 129.1, 125.4, 119.1, 86.8, 84.0, 70.0, 64.9, 64.5, 63.1, 21.5, 21.0, 20.8. HRMS: calcd for $C_{19}H_{20}O_5$ $[M+Na]^+$ 351.1203, found 351.1295. IR ($CHCl_3$): 3050, 2955, 2358, 1747, 1210 cm^{-1}. $[\alpha]_D^{23}$= -24.3^o (c = 0.5 $CHCl_3$).

Compound 3c

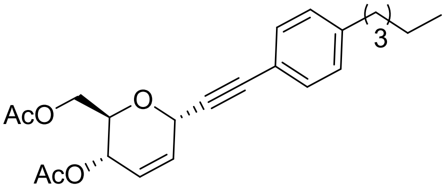

^{1}H NMR (400 MHz, $CDCl_3$) δ 7.36 (d, *J* = 8.1 Hz, 2H), 7.13 (d, *J* = 8.0 Hz, 2H), 5.98 (ddd, *J* = 10.2, 3.4, 1.8 Hz, 1H), 5.81 (d, *J* = 10.2 Hz, 1H), 5.34 (dd, *J* = 8.9, 1.8 Hz, 1H), 5.22 – 5.17 (m, 1H), 4.26 (d, *J* = 3.8 Hz, 2H), 4.23 – 4.17 (m, 1H), 2.63 – 2.57 (m, 2H), 2.11 (s, 3H), 2.10 (s, 3H), 1.60 (dt, *J* = 14.8, 7.4 Hz, 2H), 1.36 – 1.27 (m, 4H), 0.88 (t, *J* = 6.9 Hz, 3H); ^{13}C NMR (100 MHz, $CDCl_3$) δ 170.9, 170.3, 144.0, 131.7, 129.4, 128.4, 125.3, 119.3, 86.9, 83.98, 70.0, 64.8, 64.5, 63.1, 35.8, 31.4, 30.9, 22.5, 21.0, 20.8, 14.0. HRMS: calcd for $C_{23}H_{28}O_5$ $[M+Na]^+$ 407.1829, found 407.1884. IR ($CHCl_3$): 3054, 2955, 2345, 1746, 1235 cm^{-1}. $[\alpha]_D^{23}$= -37.1^o (c = 0.5 $CHCl_3$).

Compound 3d

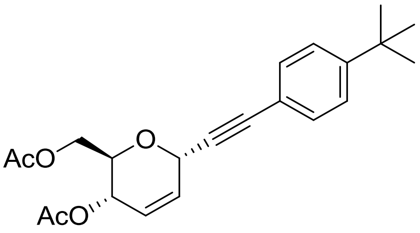

^{1}H NMR (400 MHz, $CDCl_3$) δ 7.39 (d, *J* = 8.5 Hz, 2H), 7.34 (d, *J* = 8.5 Hz, 1H), 5.98 (ddd, *J* = 10.1, 3.4, 1.8 Hz, 1H), 5.82 (d, *J* = 10.1 Hz, 1H), 5.34 (dd, *J* = 8.9, 1.9 Hz, 1H), 5.21 – 5.17 (m, 1H), 4.26 (d, *J* = 3.8 Hz, 2H), 4.23 – 4.16 (m, 1H), 2.11 (s, 3H), 2.10 (s, 3H), 1.31 (s, 6H); ^{13}C NMR (125 MHz, $CDCl_3$) δ 170.9, 170.8, 152.1, 131.7 (2C), 129.4, 125.3 (3C), 119.1, 86.8, 84.0, 69.9, 64.9, 64.6, 63.1, 31.1 (3C t_{Bu}), 29.7, 21.0, 20.8. HRMS: calcd for $C_{22}H_{26}O_5$ $[M+Na]^+$ 393.1672, found 393.1616. IR ($CHCl_3$): 3054, 2956, 2222, 1746, 1235 cm^{-1}. $[\alpha]_D^{23}$= -21.3^o (c = 0.5 $CHCl_3$)

Compound 3e

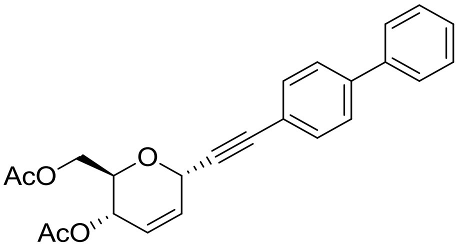

^{1}H NMR (500 MHz, $CDCl_3$) δ 7.56 (dt, *J* = 19.6, 8.2 Hz, 6H), 7.45 (dd, *J* = 8.0, 7.2 Hz, 2H), 7.40 – 7.33 (m, 1H), 6.00 (ddd, *J* = 10.2, 3.4, 1.8 Hz, 1H), 5.84 (dd, *J* = 10.2, 1.6 Hz, 1H), 5.38 – 5.32 (m, 1H), 5.23 (d, *J* = 1.6 Hz, 1H), 4.28 (d, *J* = 3.8 Hz, 2H), 4.23 (dd, *J* = 8.8, 3.8 Hz, 1H), 2.12 (d, *J* = 3.2 Hz, 6H); ^{13}C NMR (125 MHz, $CDCl_3$) δ 170.3, 169.7, 140.9, 139.5, 131.6 (2C), 128.6 (2C), 128.8, 127.1, 126.4, 126.4, 124.9, 120.4, 85.9, 84.7, 69.4, 64.2, 63.9, 62.5, 20.4, 20.2. HRMS: calcd for $C_{24}H_{22}O_5$ $[M+Na]^+$ 413.1359, found 413.1394. IR ($CHCl_3$): 3054, 2955, 2853, 1743, 1231 cm^{-1}. $[\alpha]_D^{23}$= +83.9^o (c = 0.5 $CHCl_3$).

Compound 3f

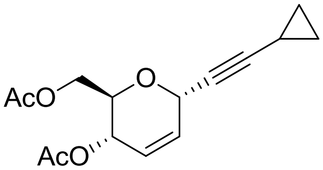

^{1}H NMR (400 MHz, $CDCl_3$) δ 5.79 (ddd, *J* = 10.2, 3.4, 1.8 Hz, 1H), 5.66 (d, *J* = 10.1 Hz, 1H), 5.21 (dd, *J* = 8.9, 1.9 Hz, 1H), 4.87 – 4.84 (m, 1H), 4.16 (d, *J* = 3.9 Hz, 2H), 4.07 – 3.98 (m, 1H), 2.04 (s, 3H), 2.02 (s, 3H), 1.35 (d, *J* = 6.0 Hz, 1H), 0.74 – 0.71 (m, 2H), 0.65 – 0.62 (m, 2H); ^{13}C NMR (100 MHz, $CDCl_3$) δ 169.5, 169.2, 128.4, 123.4, 89.3, 7.4, 68.0, 63.3, 62.7, 61.5, 60.8, 30.4, 28.2, 23.2, 21.1, 19.5, 19.3. HRMS: calcd for $C_{15}H_{18}O_5$ $[M+Na]^+$ 301.1046, found 301.1065. IR ($CHCl_3$): 2955, 2230, 1745, 1236 cm^{-1}. $[\alpha]_D^{23}$= -35.3^o (c = 0.5 $CHCl_3$).

Compound 3g

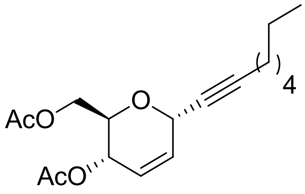

^{1}H NMR (400 MHz, $CDCl_3$) δ 5.92 – 5.85 (ddd, J= 10.14, 3.27, 1.77 Hz, 1H), 5.74 (d, *J* = 10.2 Hz, 1H), 5.33 – 5.23 (dd, J= 8.88, 1.80 Hz, 1H), 4.97 (m, 1H), 4.22 (t, *J* = 6.5 Hz, 2H), 4.16 – 4.07 (ddd, J= 8.53, 4.10 Hz, 1H, H-5), 2.28 – 2.17 (m, 2H), 2.10 (s, 3H), 2.09 (s, 3H), 1.57 – 1.45 (m, 2H), 1.45 – 1.35 (m, 2H), 1.36 – 1.26 (m, 4H), 0.89 (t, *J* = 6.8 Hz, 3H); ^{13}C NMR (100 MHz, $CDCl_3$) δ 170.9, 170.3, 130.1, 124.7, 87.8, 75.8, 69.6, 64.9, 64.2, 63.1, 31.2, 28.4, 22.5, 21.0, 20.8, 18.7, 14.0. HRMS: calcd for $C_{18}H_{26}O_5$ $[M+Na]^+$ 345.1673, found 345.1616. IR ($CHCl_3$): 3054, 2926, 2219, 1745, 1237 cm^{-1}. $[\alpha]_D^{23}$= -2.1° (c = 0.5 $CHCl_3$).

Compound 3h

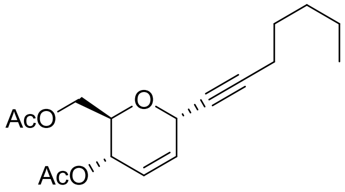

^{1}H NMR (400 MHz, $CDCl_3$) δ 5.90 – 5.86 (ddd, *J* = 10.13, 3.25, 1.77 Hz, 1H), 5.74 (d, *J* = 10.2 Hz, 1H), 5.29 (dd, *J* = 8.9, 1.9 Hz, 1H), 4.97 (m, 1H), 4.23 (d, *J* = 3.9 Hz, 2H), 4.15 – 4.09 (m, 1H), 2.22 (td, *J* = 7.1, 2.0 Hz, 2H), 2.11 – 2.09 (s, 6H), 1.55 – 1.50 (m, 2H), 1.33 (dd, *J* = 7.2, 4.6 Hz, 4H), 0.90 (t, *J* = 7.0 Hz, 3H); ^{13}C NMR (100 MHz, $CDCl_3$) δ 170.9, 170.4, 129.9, 124.7, 87.6, 75.8, 69.6, 64.89, 64.24, 63.15, 31.0, 29.7, 28.1, 22.1, 21.0, 20.8, 14.0. HRMS: calcd for $C_{18}H_{26}O_5$ $[M+K]^+$ 347.1256, found 347.1272. IR ($CHCl_3$): 3054, 2955, 2345, 1746, 1236 cm^{-1}. $[\alpha]_D^{23}$= -42.4° (c = 0.5 $CHCl_3$).

Compound 3i

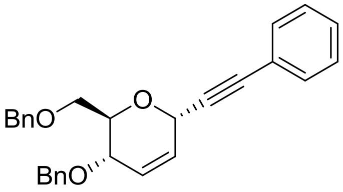

^{1}H NMR (400 MHz, $CDCl_3$) δ 7.34 – 7.27 (m, 15H), 5.99 (d, *J* = 10.2 Hz, 1H), 5.94 – 5.84 (dd, = 10.2 Hz, 1H), 5.18 (d, *J* = 1.6 Hz, 1H), 4.66 – 4.64 (m, 2H), 4.56 – 4.50 (dd, *J* = 11.9 Hz, 2H), 4.19 (dd, *J* = 9.0, 1.7 Hz, 1H), 4.04 (ddt, *J* = 9.2, 3.8 Hz, 11H), 3.79 – 3.75 (d, *J* = 2.0 Hz, 2H). 13C NMR (100 MHz, CDCl3) δ 138.15, 137.99, 131.87, 128.52, 128.44, 128.36, 128.25, 128.03 (3 x C), 127.86, 127.80, 127.65, 127.01, 122.49, 86.14, 85.69, 73.41, 72.30, 71.41, 69.94, 68.93, 64.62. HRMS: calcd for $C_{28}H_{26}O_3$ $[M+H]^+$ 411.1955, found 411.1994. IR ($CHCl_3$): 3087, 2954, 2345, 1728 cm^{-1}. $[\alpha]_D^{23}$= +11.7^o (c = 0.5 $CHCl_3$).

Compound 3j

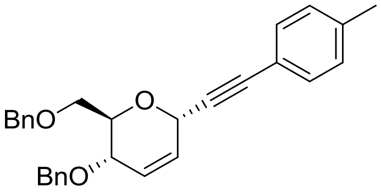

^{1}H NMR (500 MHz, $CDCl_3$) δ 7.31 (tdd, *J* = 13.1, 10.2, 6.5 Hz, 12H), 7.10 (d, *J* = 7.8 Hz, 2H), 5.99 (d, *J* = 10.2 Hz, 1H), 5.90 (ddd, *J* = 10.2, 3.3, 1.7 Hz, 1H), 5.18 (d, *J* = 1.4 Hz, 1H), 4.64 (t, *J* = 12.2 Hz, 2H), 4.54 (d, *J* = 12.2 Hz, 1H), 4.47 (d, *J* = 11.4 Hz, 1H), 4.22 (dd, *J* = 8.9, 1.7 Hz, 1H), 4.10 – 4.03 (m, 1H), 3.77 (d, *J* = 3.0 Hz, 2H), 2.34 (s, 3H); ^{13}C NMR (125 MHz, $CDCl_3$) δ 136.7, 136.2, 136.0, 129.8 (2C), 127.0 (2C), 126.8, 126.4, 126.0 (4C), 125.9, 125.8, 125.7, 124.9, 117.4, 84.3, 83.0, 71.4, 70.2, 69.4, 67.9, 66.9, 62.7, 19.5. HRMS: calcd for $C_{29}H_{28}O_3$ $[M+Na]^+$ 463.1671, found 463.1680. IR ($CHCl_3$): 3063, 2955, 2219, 1728, 1207 cm^{-1}. $[\alpha]_D^{23}$= -8.7^o (c = 0.5 $CHCl_3$).

Compound 3k

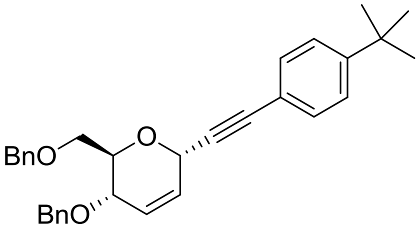

^{1}H NMR (500 MHz, $CDCl_3$) δ 7.41 – 7.29 (m, 14H), 6.01 (d, *J* = 10.2 Hz, 1H), 5.94 – 5.89 (ddd, *J* = 10.3, 3.4, 1.7 Hz, 1H), 5.20 (d, *J* = 1.4 Hz, 1H), 4.71 – 4.61 (m, 2H), 4.54 (dd, *J* = 11.2 Hz, 2H), 4.27 – 4.22 (m, 1H), 4.13 – 4.07 (m, 1H), 3.83 – 3.76 (m, 2H), 1.31 (d, *J* = 15.5 Hz, 10H); ^{13}C NMR (125 MHz, $CDCl_3$) δ 151.8, 138.2, 138.1, 31.6 (2C), 128.4, 128.3(4C), 128.0 (4C), 187.0, 127.8, 127.6, 126.9, 125.2, 119.5, 8.2, 85.0, 73.4, 72.2, 71.3, 70.0, 68.9, 64.7, 31.1. HRMS: calcd for $C_{32}H_{34}O_3$ $[M+K]^+$ 505.2140, found 505.2209. IR ($CHCl_3$): 3087, 2957, 2198, 1746, 1268 cm^{-1}. $[\alpha]_D^{23}$= -58.2° (c = 0.5 $CHCl_3$). $[\alpha]_D^{23}$= -101.3° (c = 0.5 $CHCl_3$).

Compound 3l

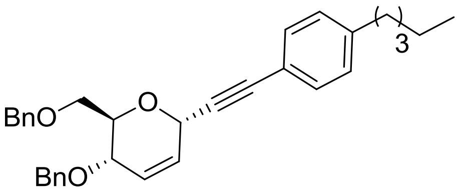

^{1}H NMR (400 MHz, $CDCl_3$) δ 7.39 – 7.26 (m, 12H), 7.11 (t, *J* = 13.9 Hz, 2H), 5.98 (d, *J* = 10.2 Hz, 1H), 5.93 – 5.84 (ddd, J = 10.2, 3.3, 1.7 Hz, 1H), 5.27 – 5.10 (bs, 1H), 4.62 (td, *J* = 11.9, 7.8 Hz, 2H), 4.51 (dd, *J* = 26.1, 11.8 Hz, 2H), 4.21 (dd, *J* = 8.9, 1.7 Hz, 1H), 4.07 (dt, *J* = 8.8, 3.0 Hz, 1H), 3.77 (d, *J* = 3.0 Hz, 2H), 2.64 – 2.51 (m, 2H), 1.62-1.55 (dt, *J* = 15.1, 7.7 Hz, 2H), 1.37 – 1.27 (m, 4H), 0.88 (t, *J* = 6.9 Hz, 3H); ^{13}C NMR (100 MHz, $CDCl_3$) δ 143.7, 138.2, 138.0, 131.80 (2C), 128.4 (2C), 128.4 (2C), 128.3, 128.0, 128.0 (6C), 127.8, 126.9, 119.6, 86.3, 85.0, 73.4, 72.2, 71.4, 69.9, 68.9, 64.7, 35.8, 31.4, 30.9, 22.5, 14.0. HRMS: calcd for $C_{33}H_{36}O_3$ $[M+Na]^+$ 503.2557, found 503.2592. IR ($CHCl_3$): 3086, 2954, 2218, 1738, 1277, 1094 cm^{-1}. $[\alpha]_D^{23}$= -18.1° (c = 0.5 $CHCl_3$).

Compound 3m

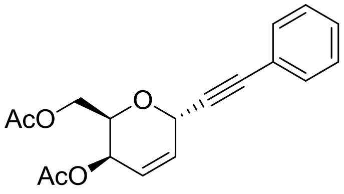

^{1}H NMR (500 MHz, $CDCl_3$) δ 7.50 – 7.41 (dt, *J* = 8.4, 3.7 Hz, 2H), 7.38 – 7.29 (m, 3H), 6.15 (dd, *J* = 10.0, 3.7 Hz, 1H), 6.11 – 6.02 (ddd, J = 10.1, 5.3, 1.8 Hz, 1H), 5.28 (dd, J = 3.6, 1.7 Hz, 1H), 5.18 – 5.08 (dd, J = 5.4, 2.3 Hz, 1H), 4.50 – 4.42 (ddd, J = 7.4, 5.2, 2.4 Hz, 1H), 4.32 (dd, *J* = 11.5, 5.2 Hz, 1H), 4.22 (dd, *J* = 11.5, 7.4 Hz, 1H), 2.10 (s, 3H), 2.08 (s, 3H). ^{13}C NMR (125 MHz, $CDCl_3$) δ 170.8, 170.4, 132.0, 131.8, 128.8, 128.4, 122.4, 122.1, 86.9, 84.1, 69.71, 64.4, 63.3, 62.8, 20.9, 20.8. HRMS: calcd for $C_{18}H_{18}O_5$ $[M+Na]^+$ 337.1047, found 337.1059. IR ($CHCl_3$): 3054, 2926, 2220, 1746, 1235 cm^{-1}. $[\alpha]_D^{23}$= -189.3^o (c = 0.5 $CHCl_3$).

Compound 3n

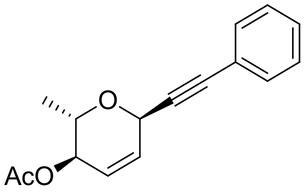

^{1}H NMR (400 MHz, $CDCl_3$) δ 7.45 (dt, *J* = 8.4, 3.7 Hz, 2H), 7.35 – 7.27 (m, 3H), 5.95 (ddd, *J* = 10.2, 3.3, 1.8 Hz, 1H), 5.82 – 5.76 (m, 1H), 5.13 (dd, *J* = 3.2, 1.7 Hz, 1H), 5.07 (ddd, *J* = 8.2, 3.9, 1.9 Hz, 1H), 4.08 (dq, *J* = 12.6, 6.3 Hz, 1H), 2.09 (s, 3H), 1.29 (d, *J* = 6.3 Hz, 3H). ^{13}C NMR (100 MHz, $CDCl_3$) δ 170.5, 131.8, 129.3, 128.6, 128.3, 125.7, 122.4, 86.0, 85.6, 70.3, 68.2, 63.8, 21.1, 18.1. HRMS: calcd for $C_{16}H_{16}O_3$ $[M+Na]^+$ 279.0992, found 279.0985. IR ($CHCl_3$): 3054, 2976, 2221, 1744, 1234 cm^{-1}. $[\alpha]_D^{23}$= -15.8^o (c = 0.5 $CHCl_3$).

NMR Spectra's

^{1}H NMR of 3a

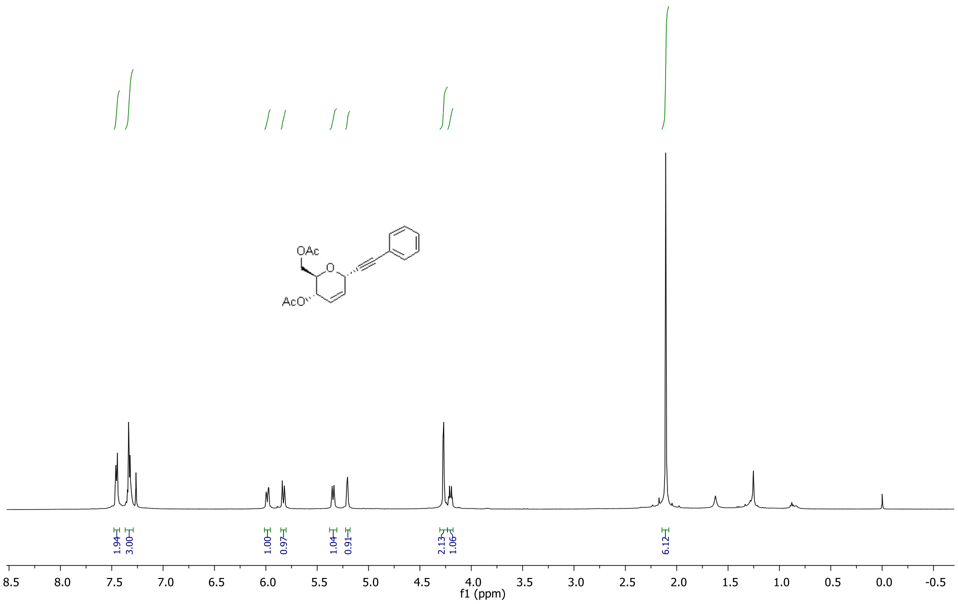

^{13}C NMR of 3a

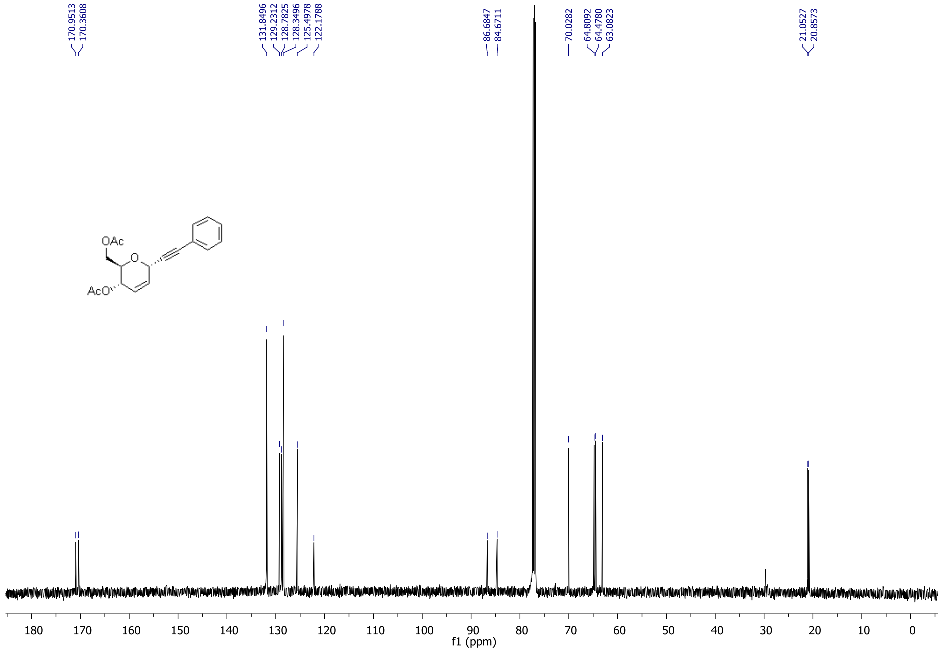

NOESY spectra of compound 3a

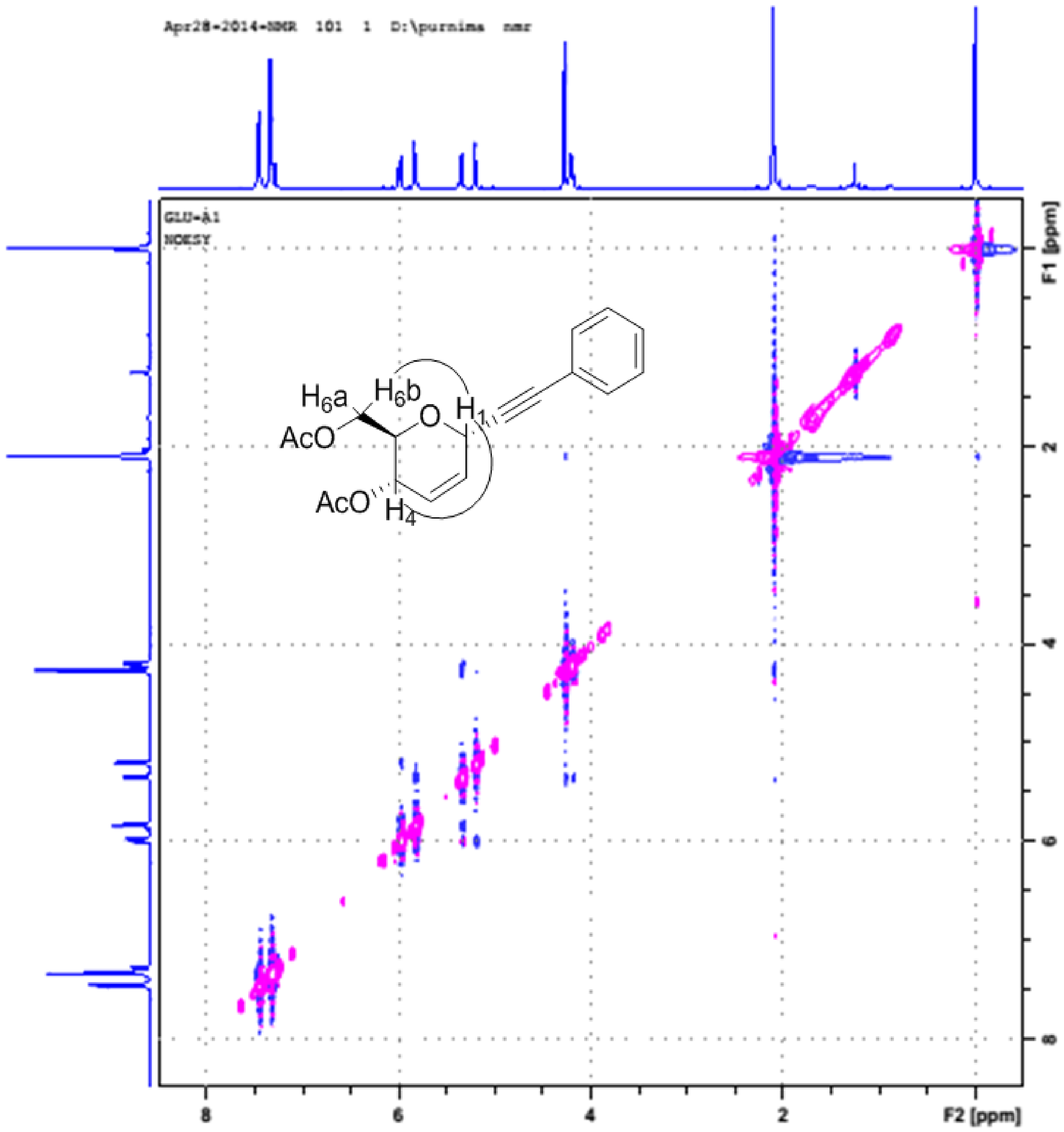

^{1}H NMR of 3b

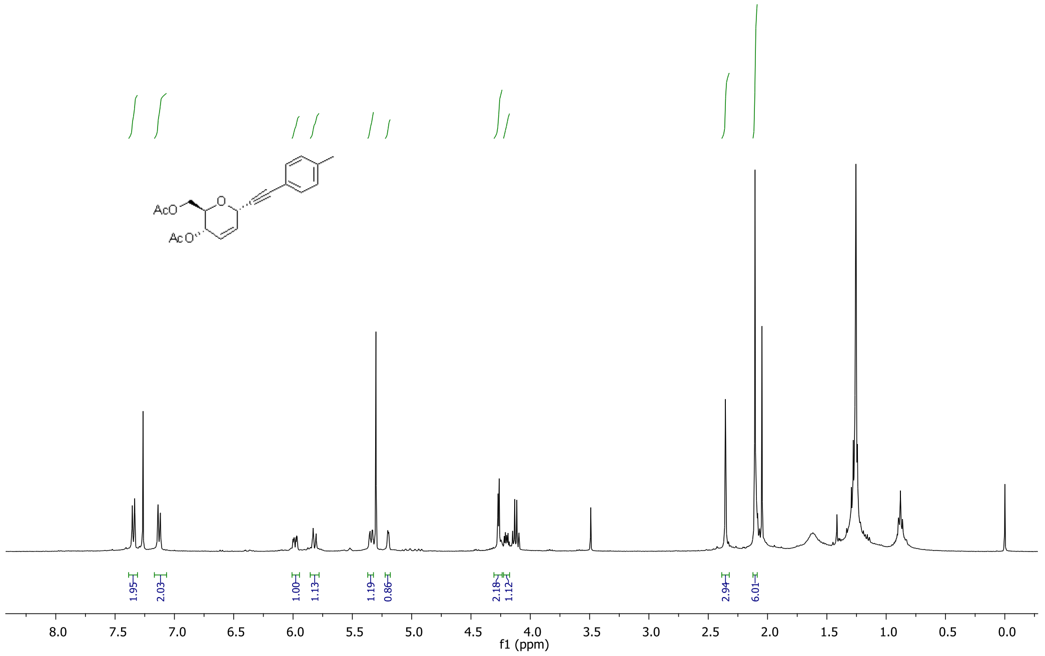

^{13}C NMR of 3b

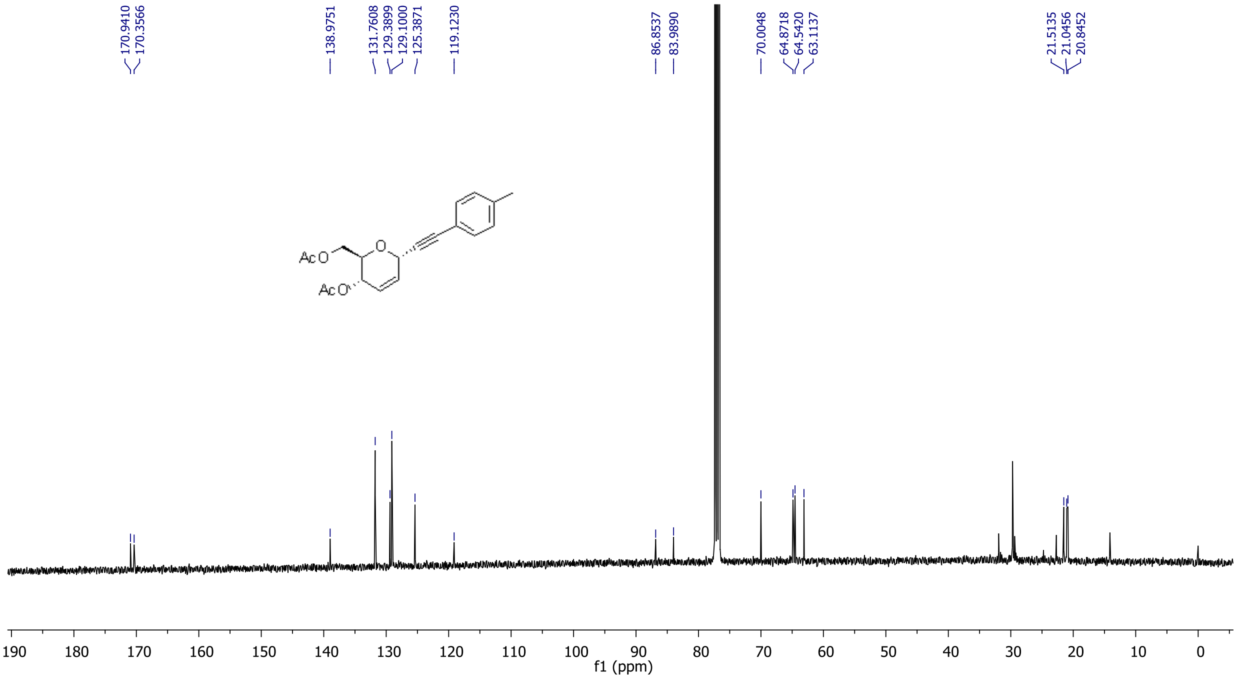

^{1}H NMR of 3c

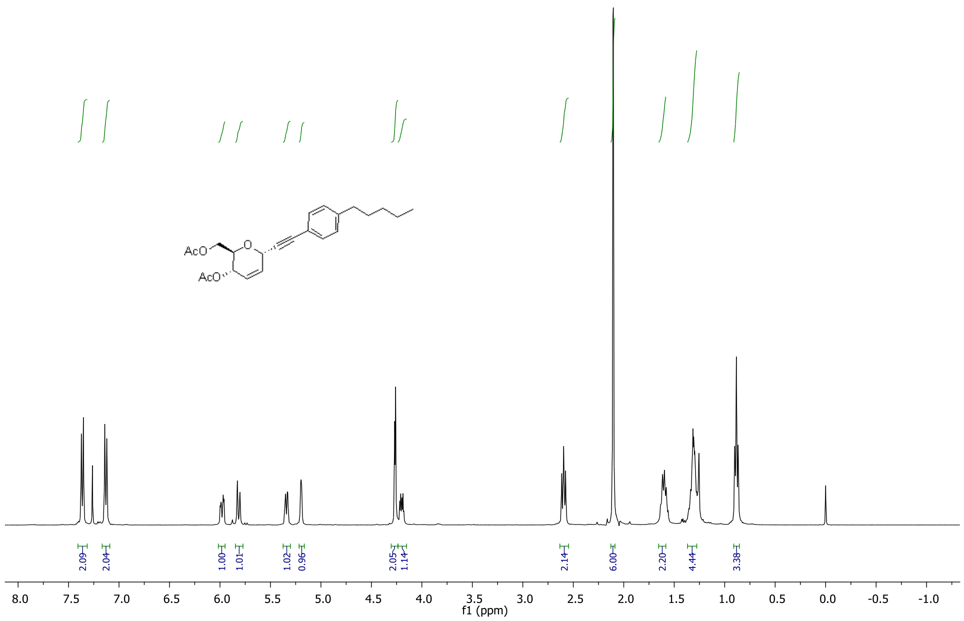

C[13] NMR of 3c

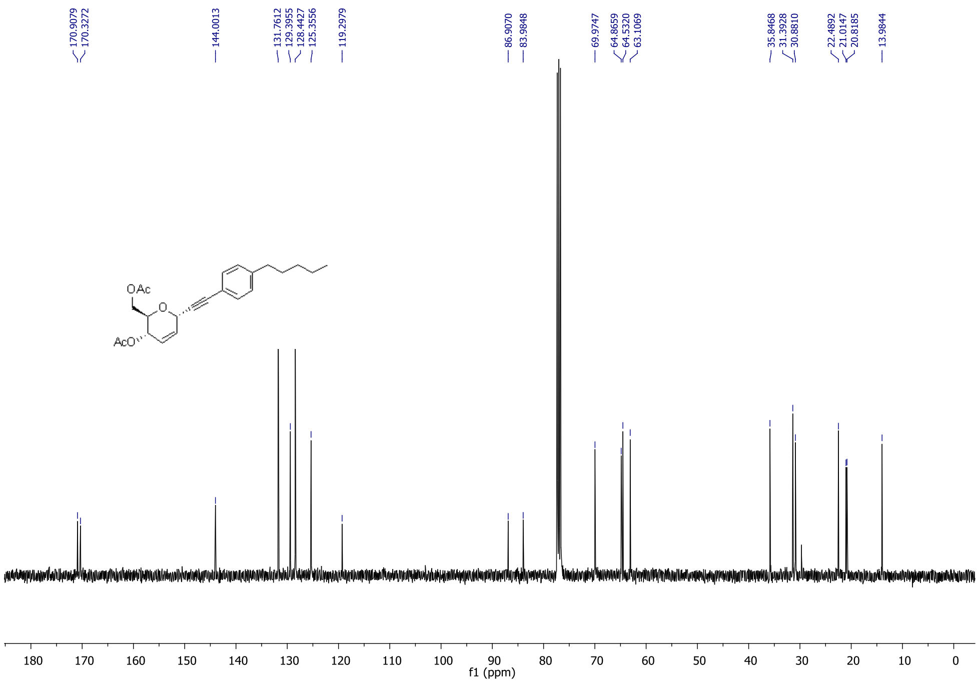

^{1}H NMR of 3d

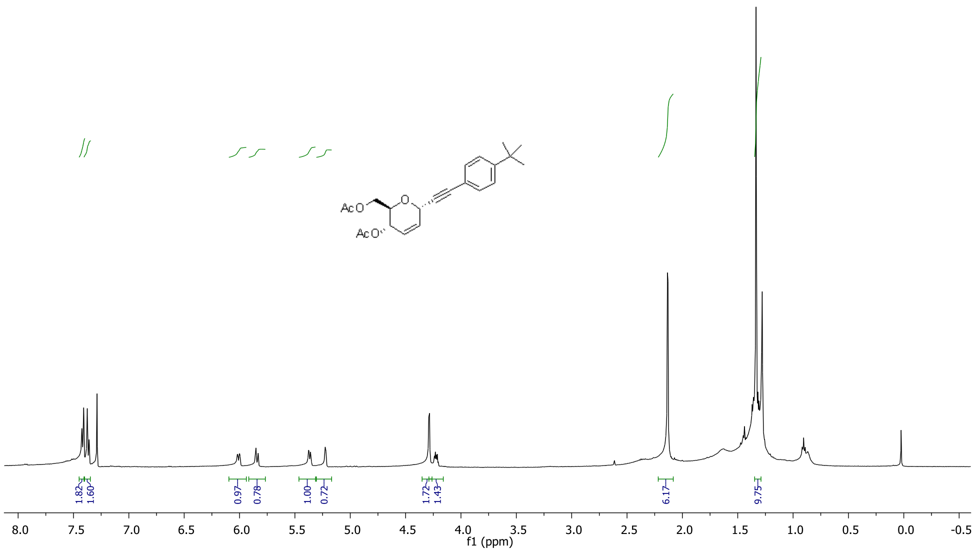

^{13}C NMR of 3d

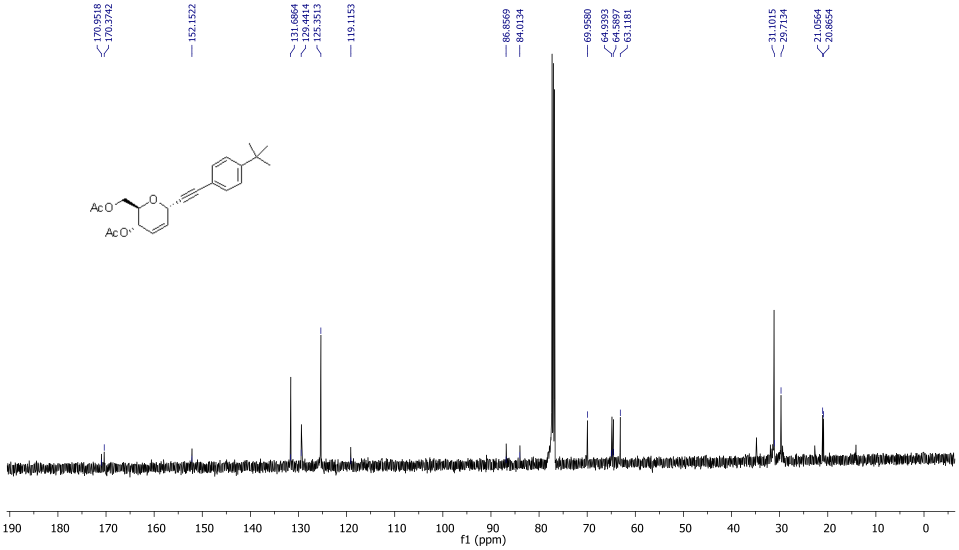

^{1}H NMR of 3e

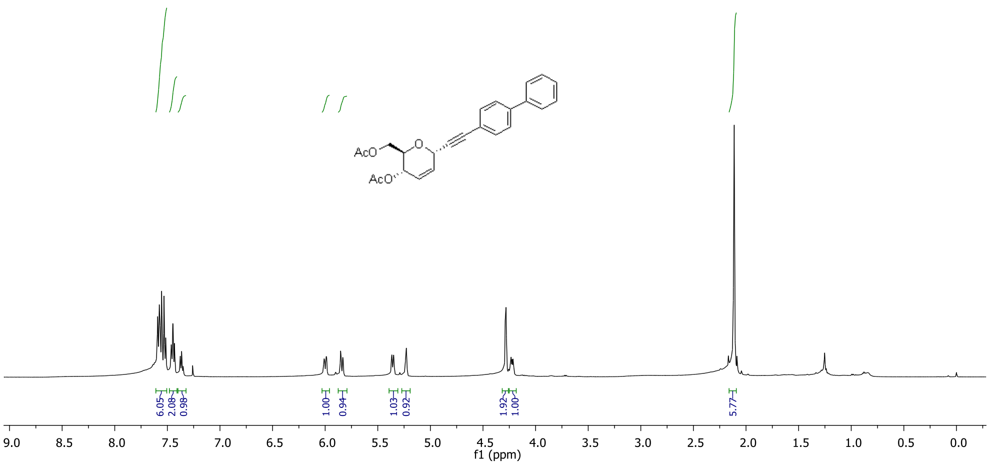

^{13}C NMR of 3e

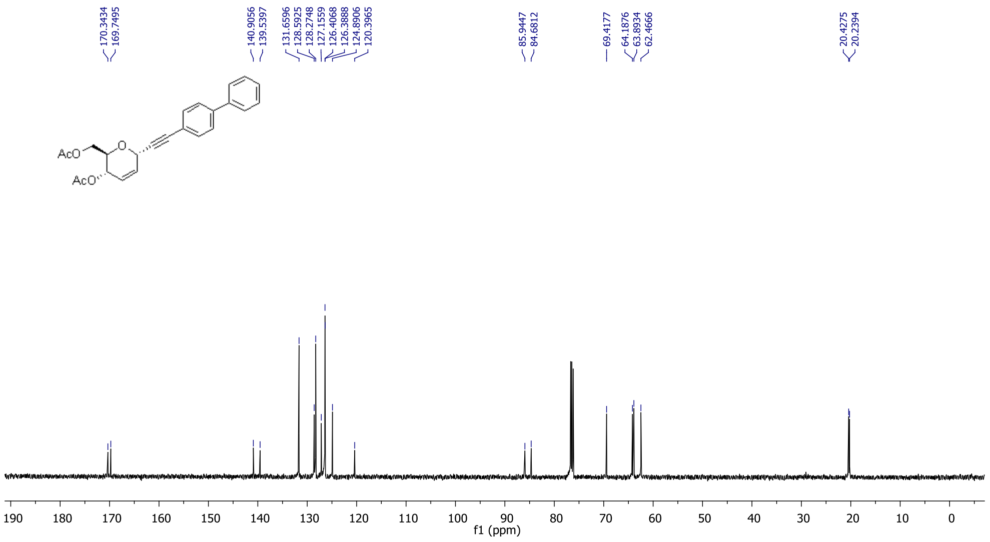

^{1}H NMR of 3f

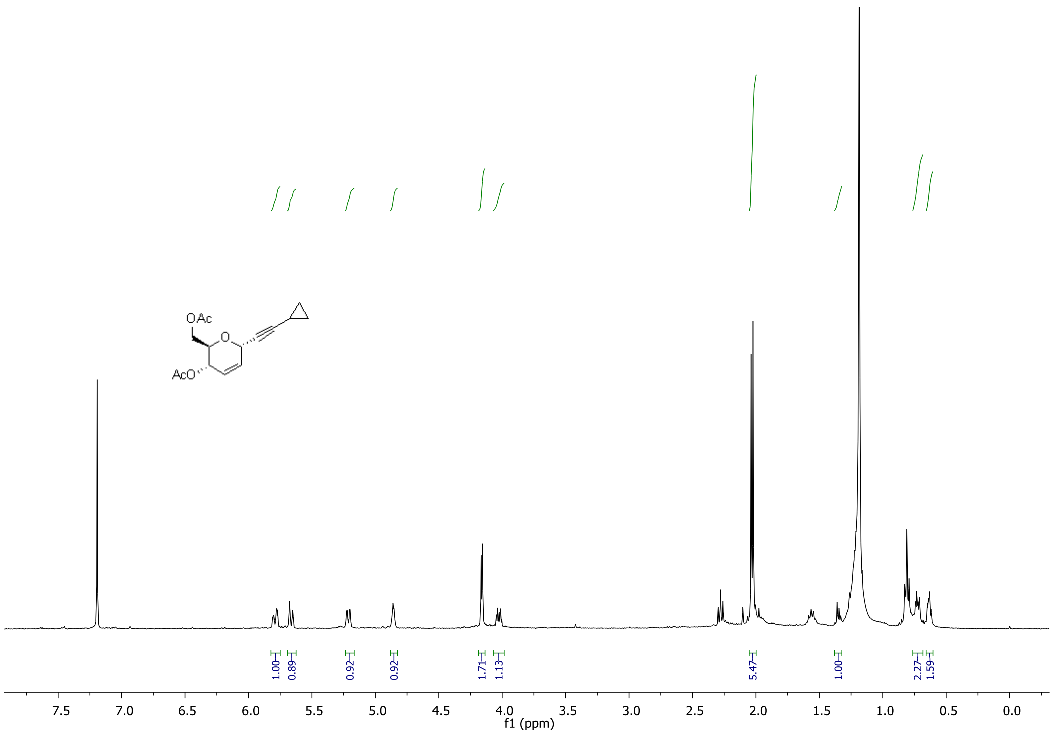

^{13}C NMR of 3f

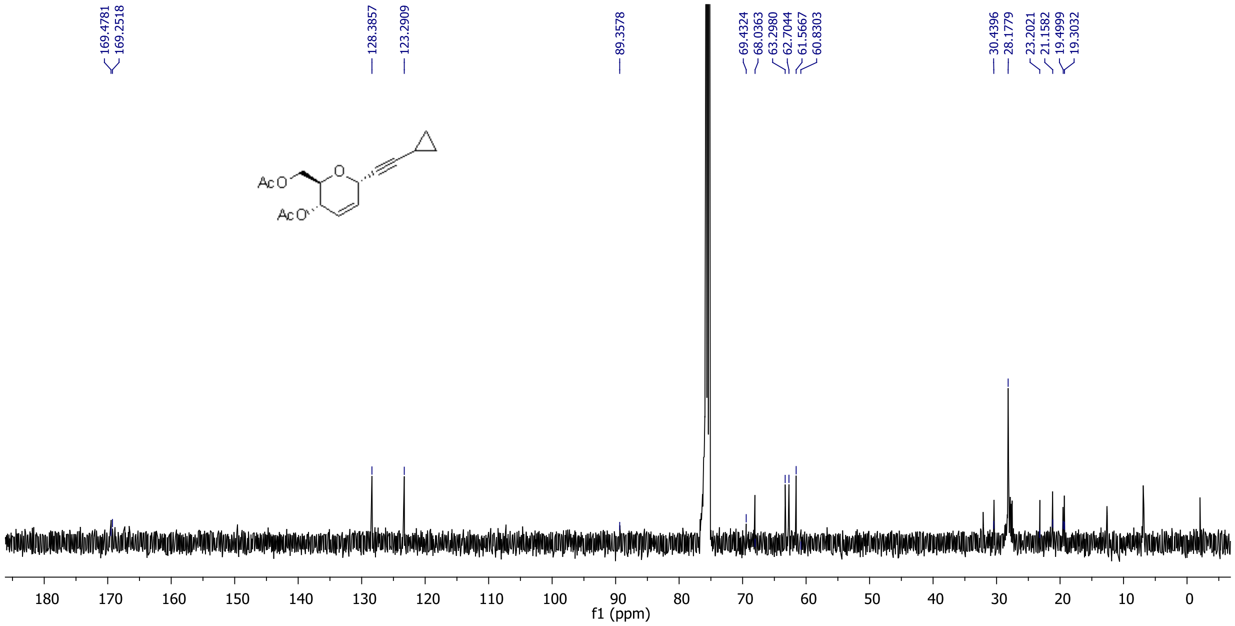

^{1}H NMR of 3g

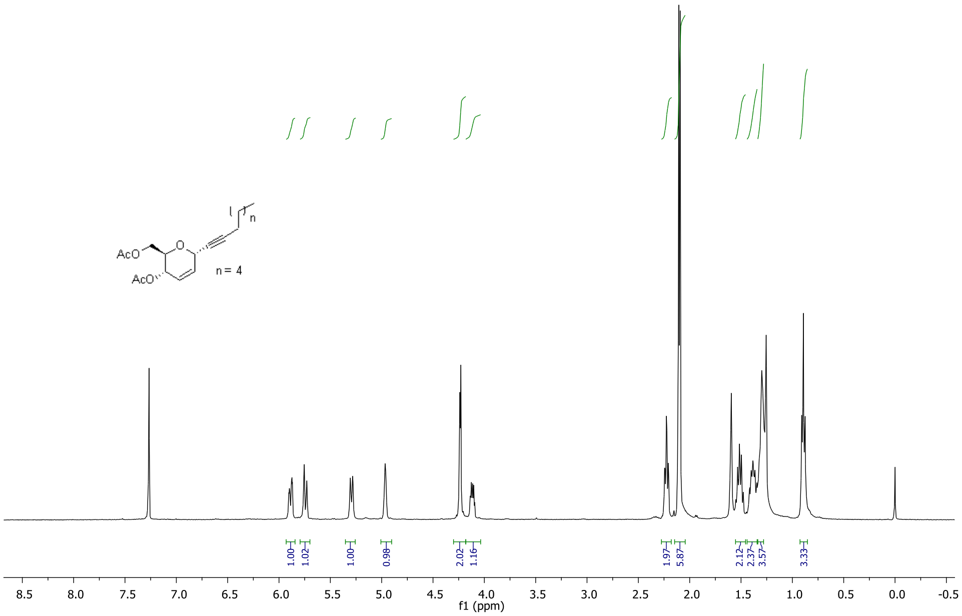

^{13}C NMR of 3g

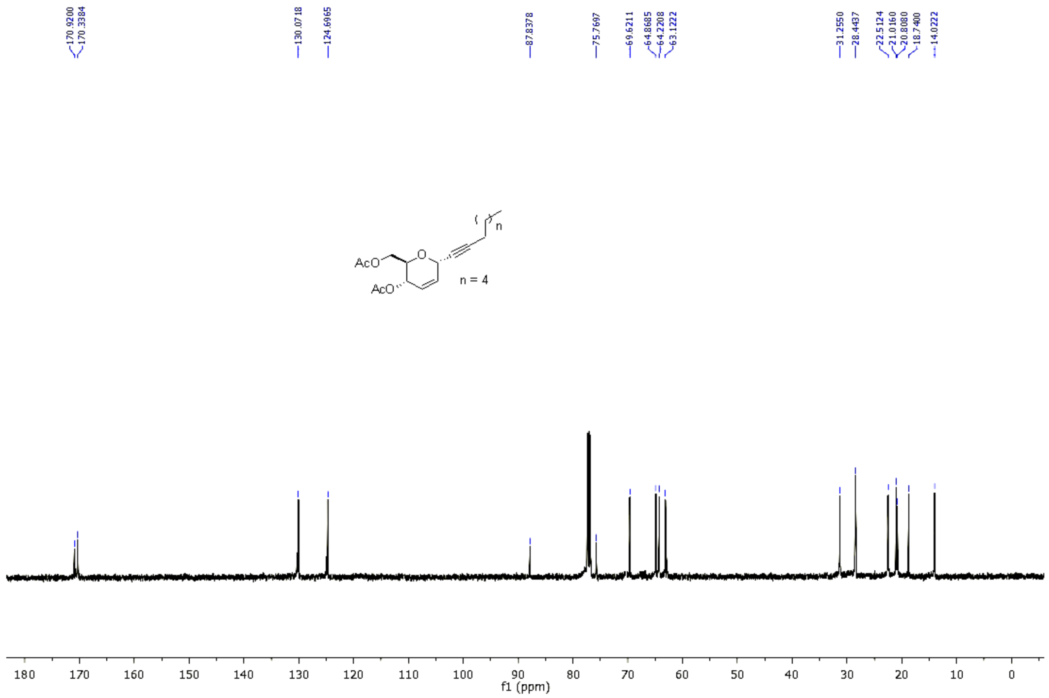

^{1}H NMR of 3h

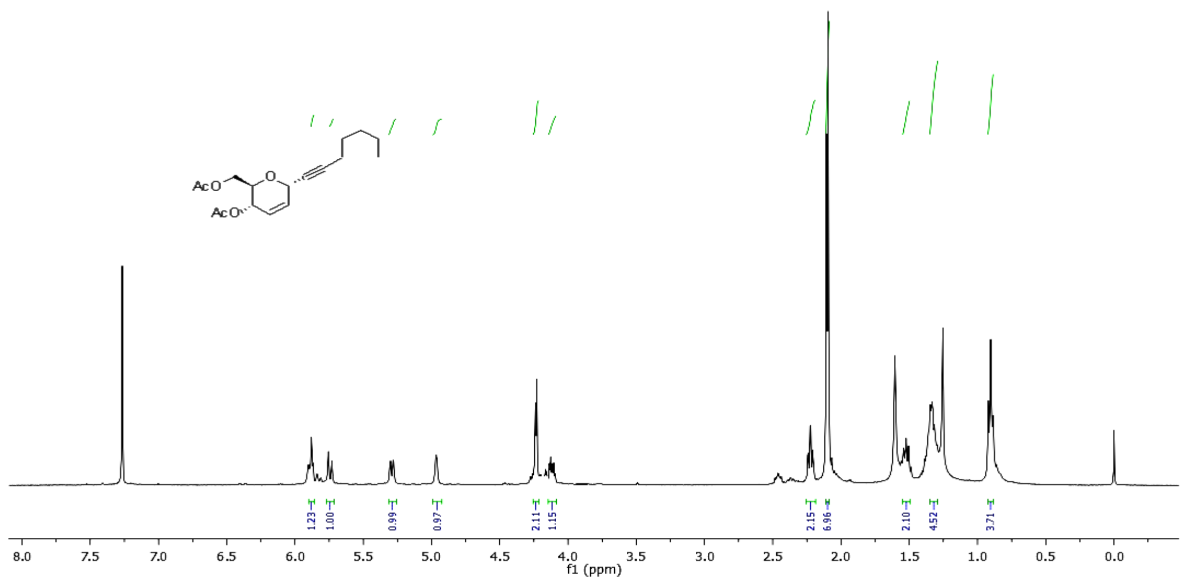

^{13}C NMR of 3h

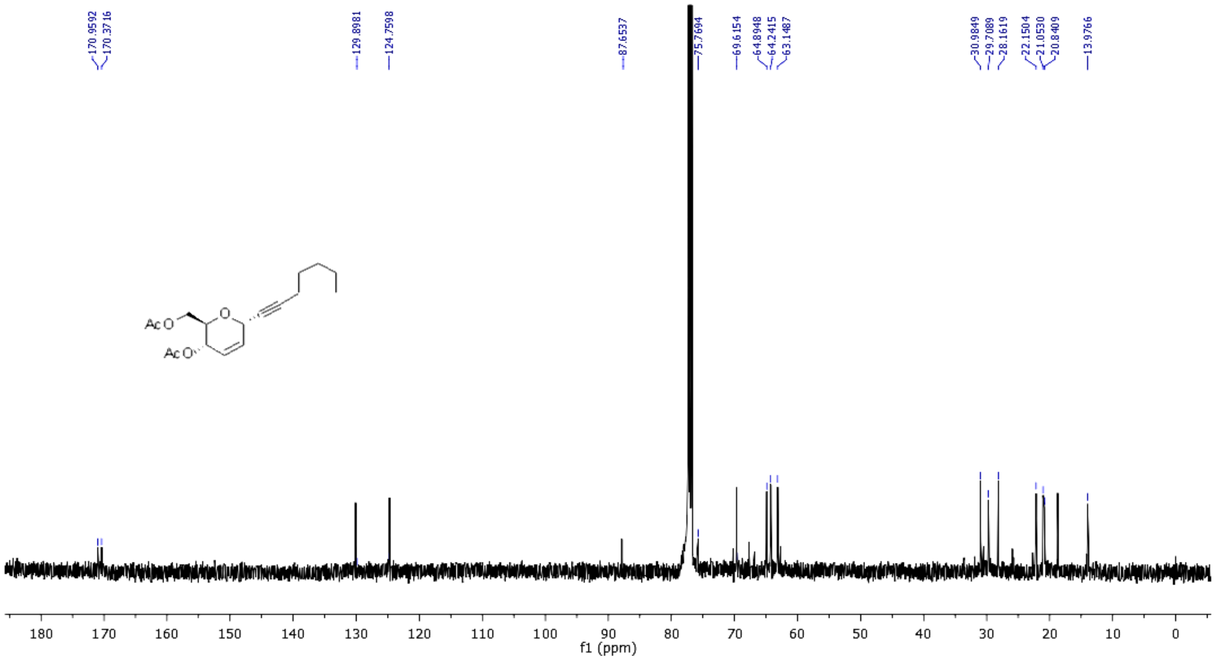

^{1}H NMR of 3i

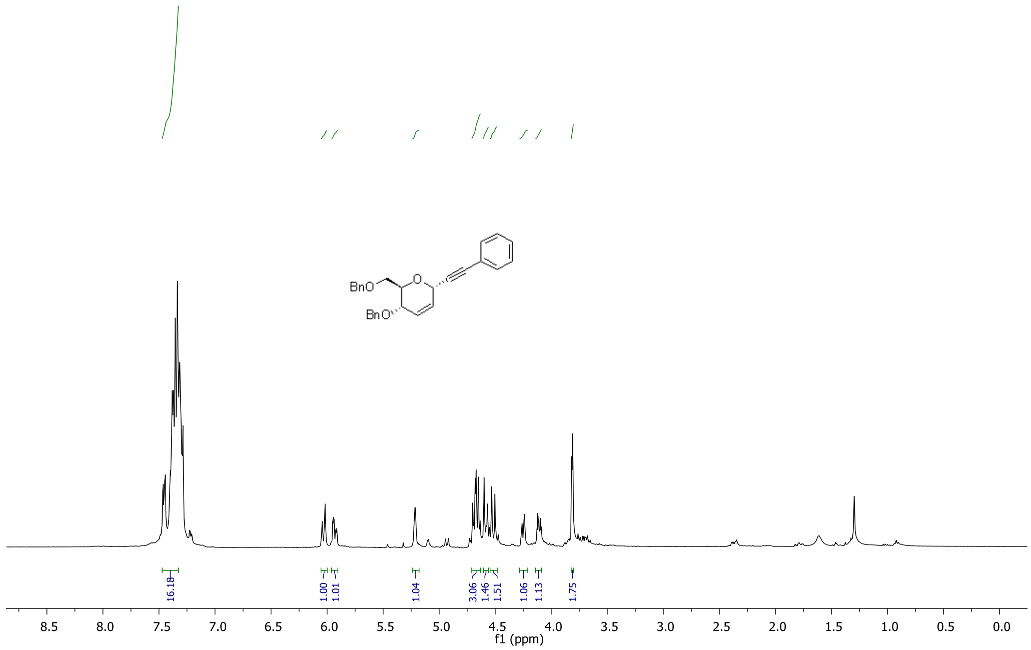

^{13}C NMR of 3i

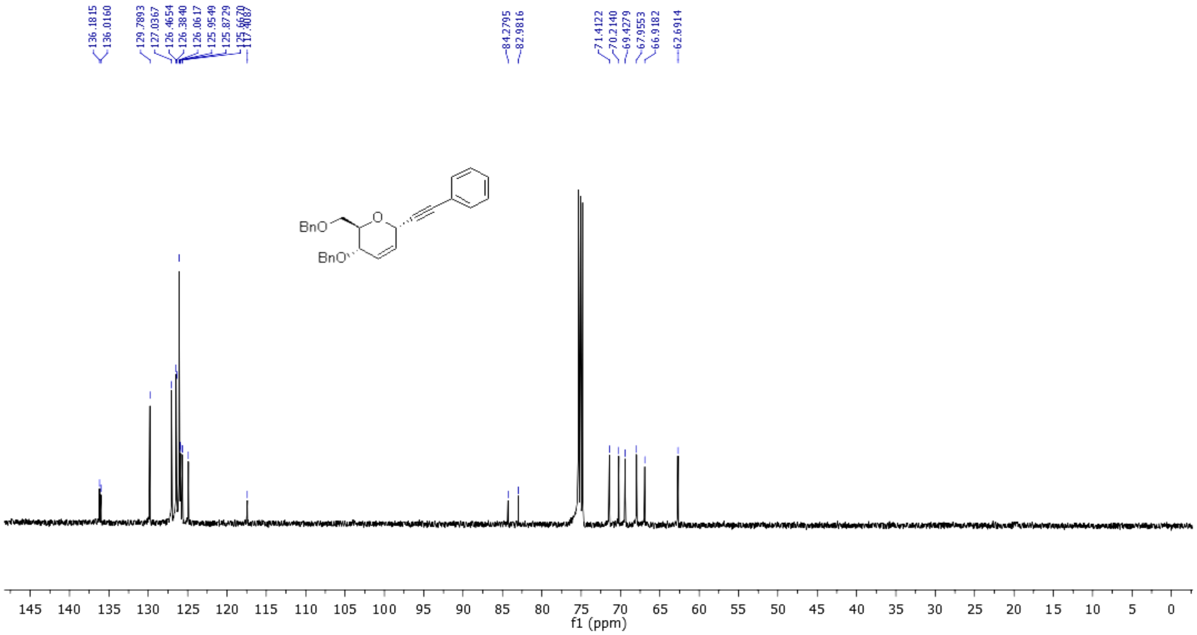

^{1}H NMR of 3j

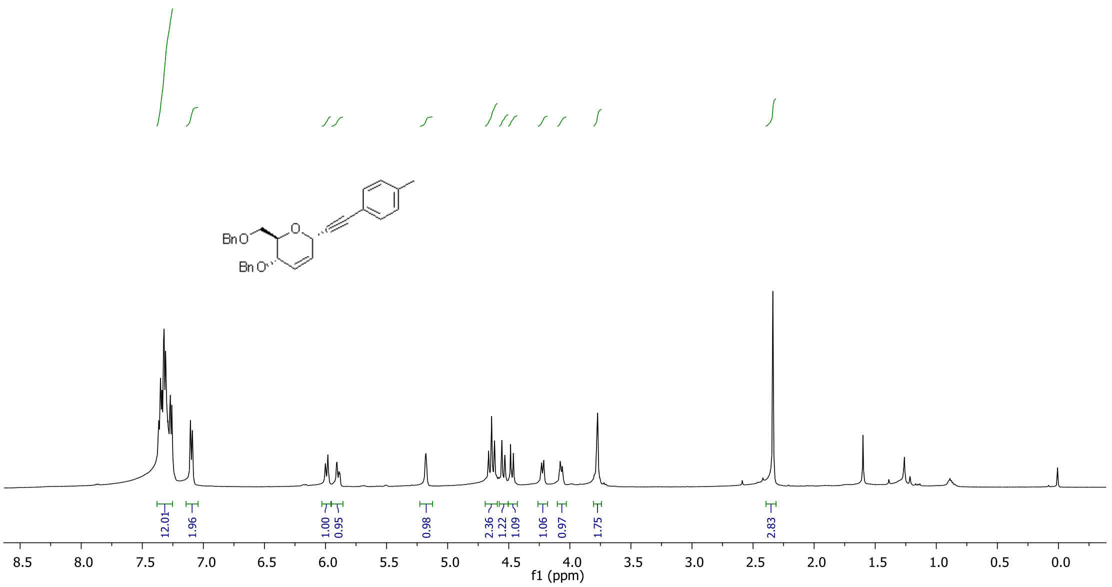

^{13}C NMR of 3j

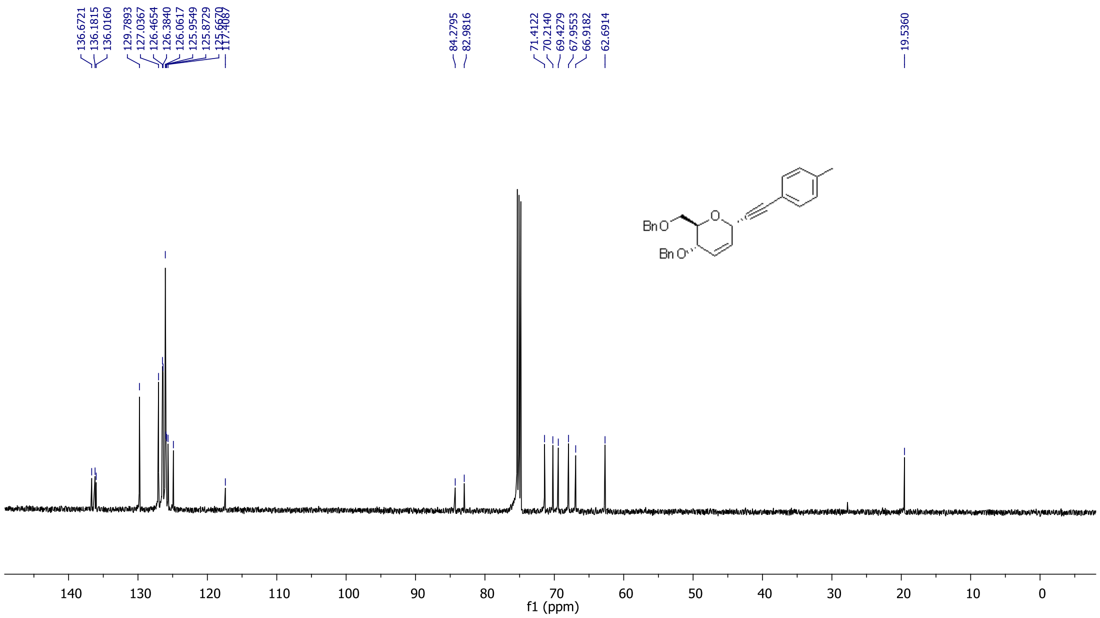

^{1}H NMR of 3k

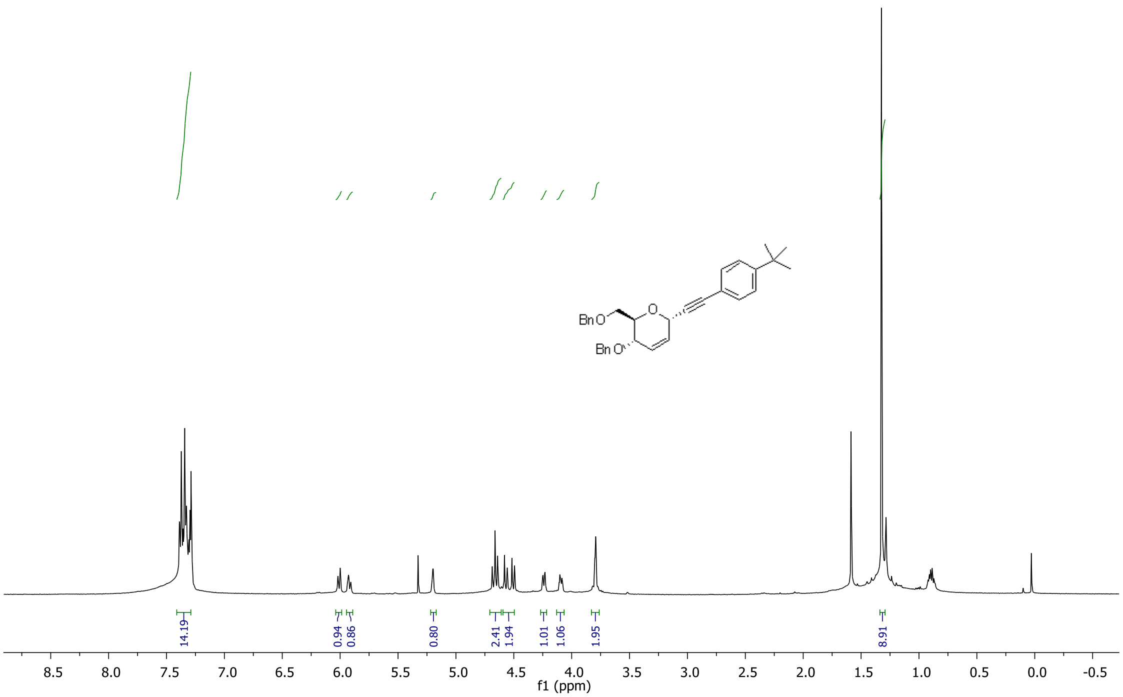

^{13}C NMR of 3k

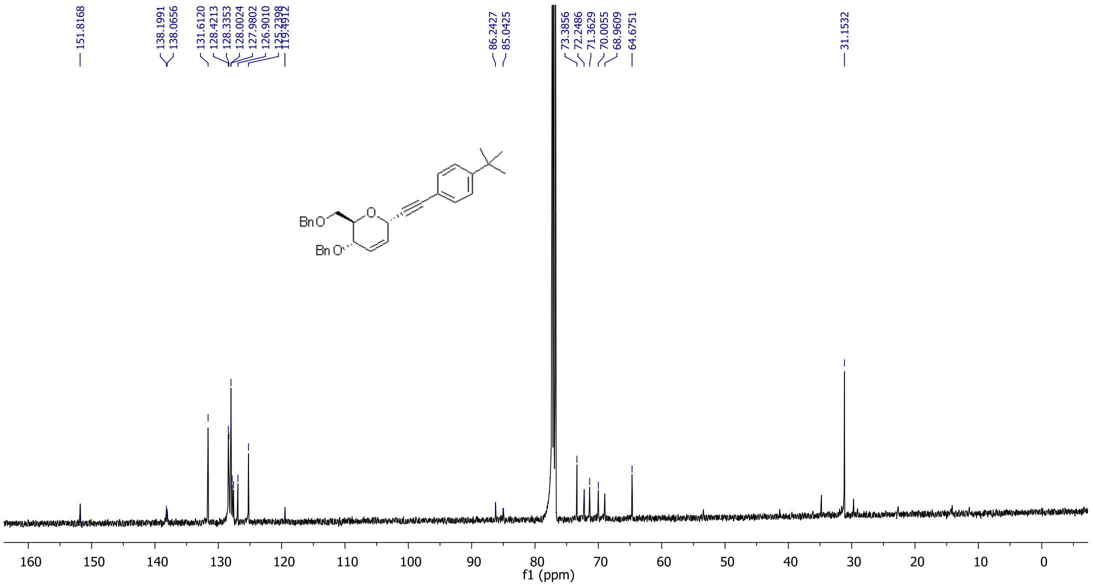

^{1}H NMR of 3l

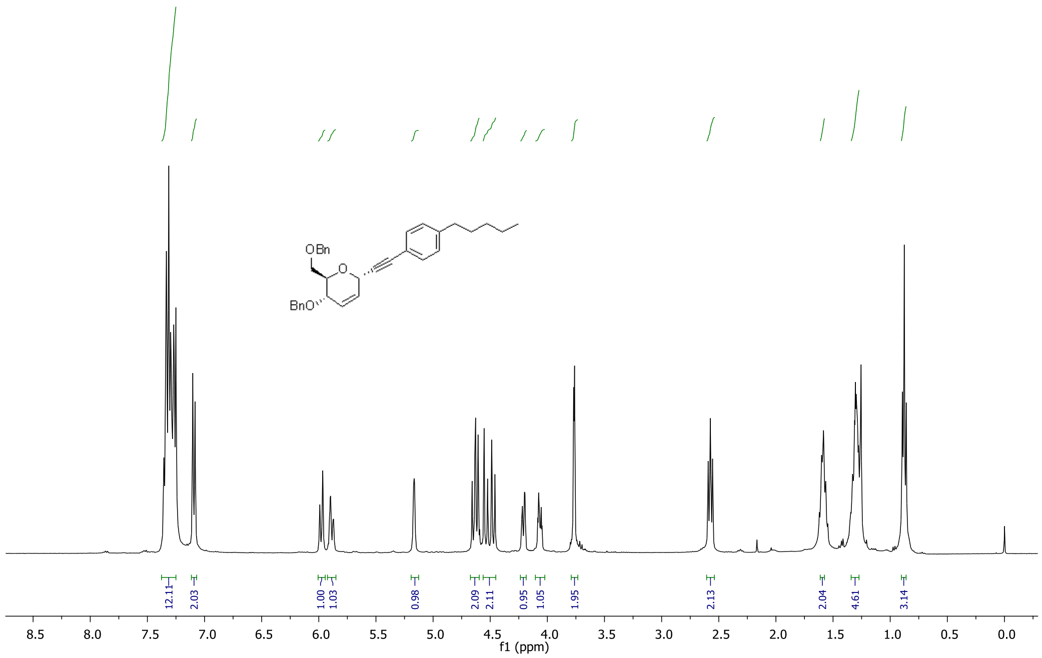

^{13}C NMR of 3l

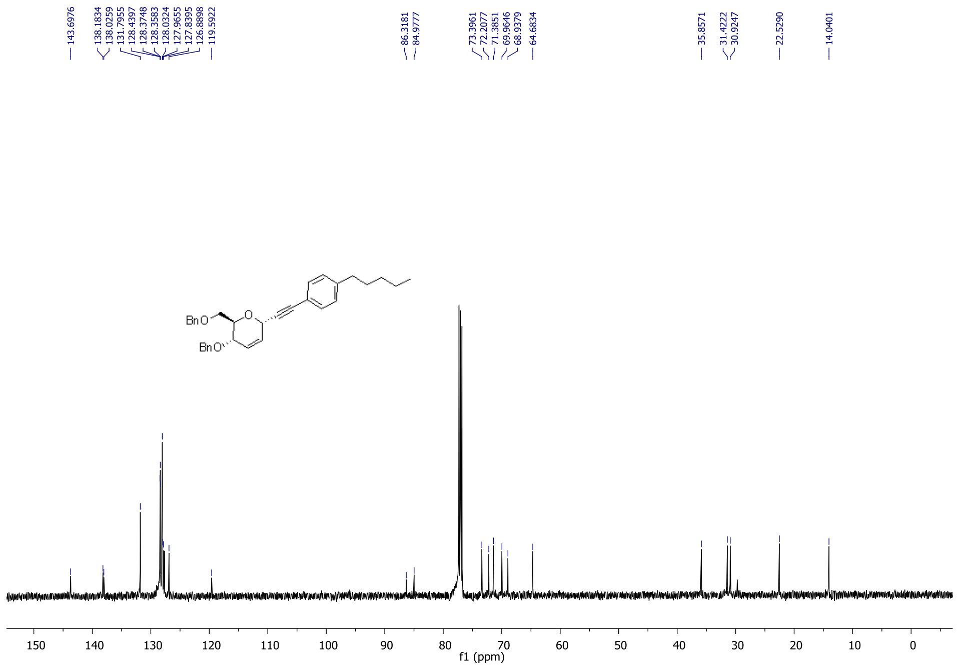

^{1}H NMR of 3m

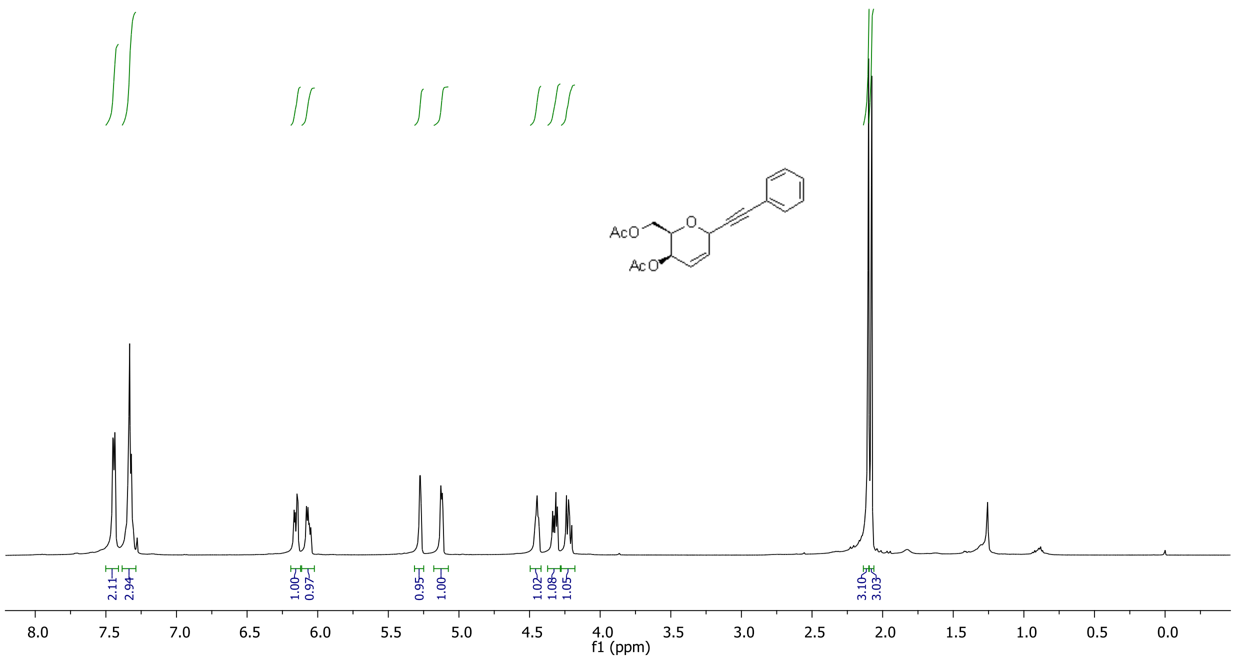

^{13}C NMR of 3m

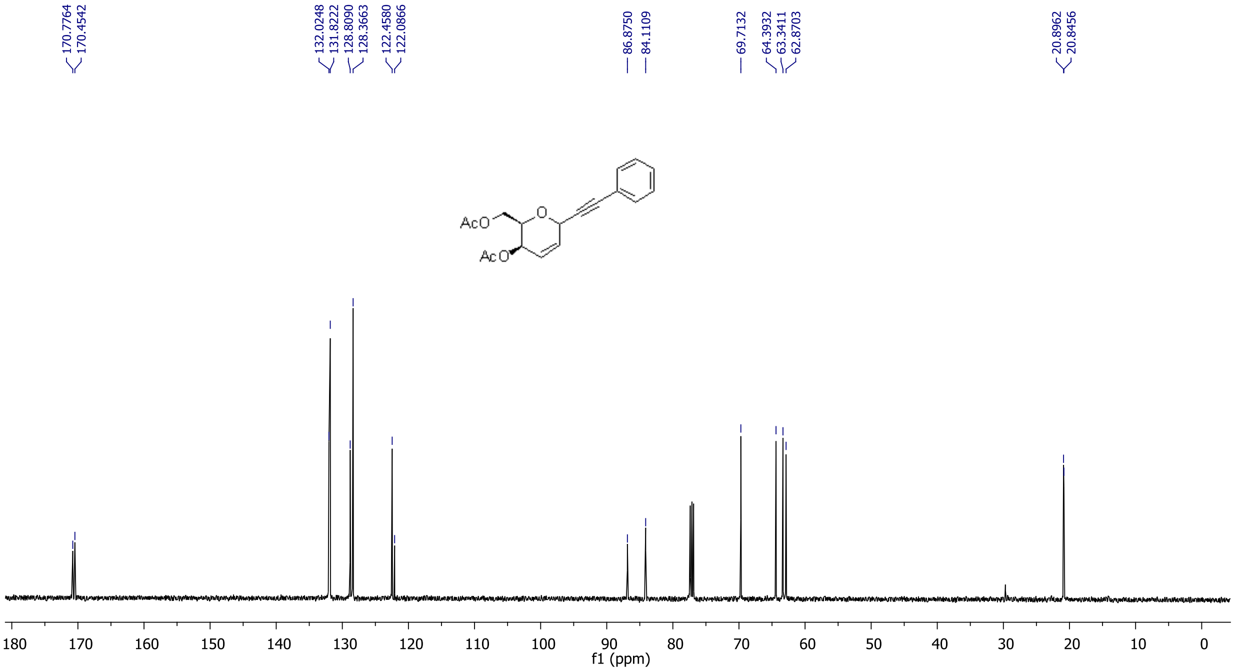

^{1}H NMR of 3n

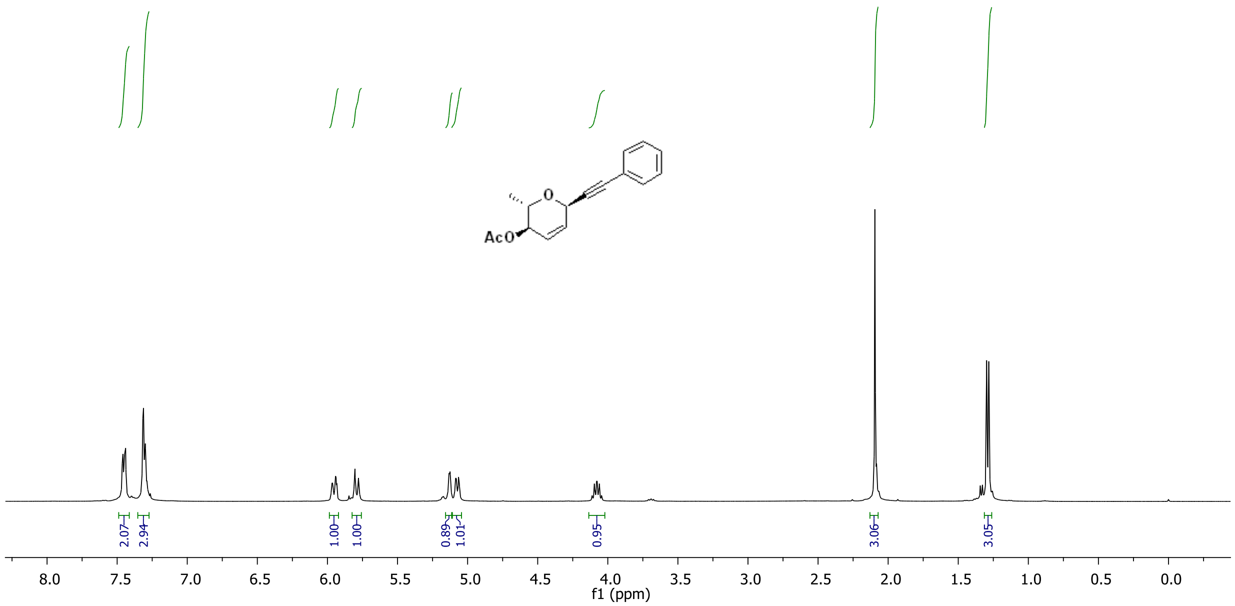

^{13}C NMR of 3n

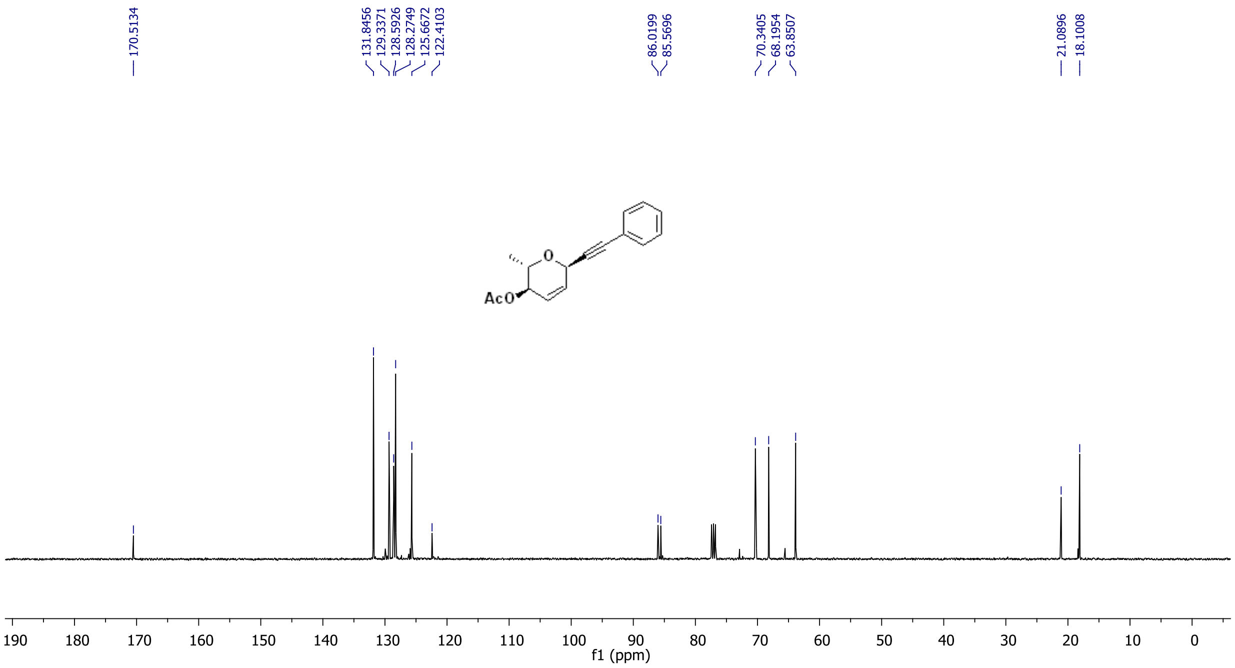

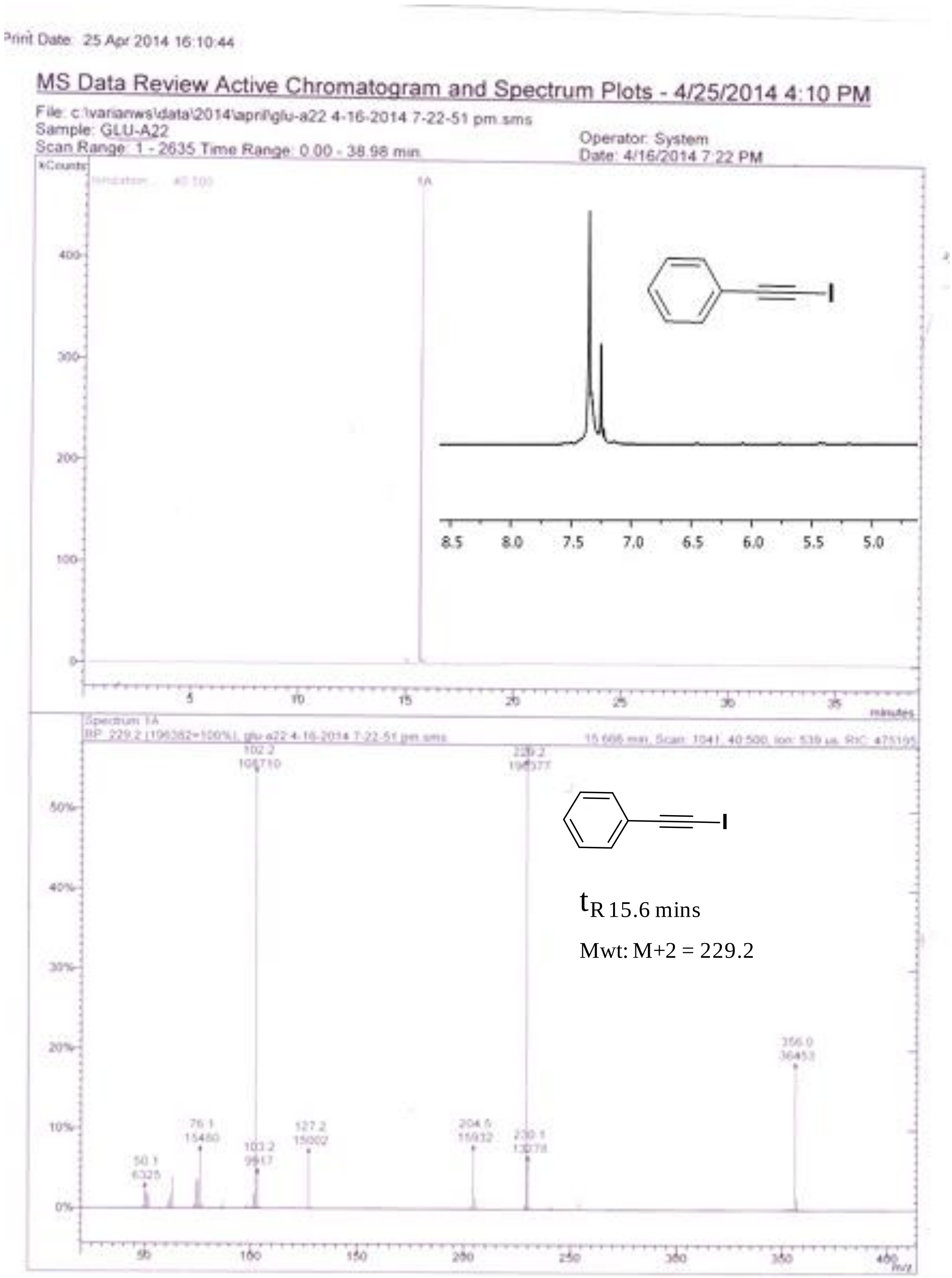

Figure S1: ^{1}H NMR and GC–MS spectra of iodinated phenylacetylene.
